# Detection of malaria parasites in samples from returning US travelers using the Alethia® Malaria Plus LAMP assay

**DOI:** 10.1186/s13104-021-05542-7

**Published:** 2021-04-07

**Authors:** Dragan Ljolje, Rispah Abdallah, Naomi W. Lucchi

**Affiliations:** Malaria Branch, Division of Parasitic Diseases and Malaria, Center for Global Health, Centers for Disease Control and Prevention, Atlanta, GA USA

**Keywords:** Malaria, Plasmodium, LAMP, Molecular diagnostics, Imported malaria

## Abstract

**Objective:**

In this study, the performance of a commercially available malaria LAMP assay (Alethia® Malaria Plus LAMP) was evaluated using retrospective clinical samples obtained from travelers returning to the United States of America (USA). Recently, several laboratories in non-malaria endemic countries evaluated the use of the loop mediated isothermal amplification (LAMP) assays for the diagnosis of imported malaria cases. These tests are simpler than polymerase-chain reaction (PCR)-based assays and were shown to have high sensitivity. Much of malaria diagnoses in the USA, is undertaken at the state level using mainly microscopy and rapid diagnostic tests (RDTs). However, molecular tools offer greater sensitivity over microscopy and RDTs. A reliable, easy to perform molecular assay can provide a test of choice for the accurate detection of malaria parasites in places where expert microscopy is lacking and/or for the detection of low-parasite density infections.

**Results:**

The Alethia® Malaria Plus LAMP assay was easy to use, had similar test performances as the real-time PCR reference test and results were obtained faster (within 1 h) than the reference test. The sensitivity of the assay was 100% with a kappa score of 1 when compared to the reference PET-PCR assay.

## Introduction

Malaria is a disease of the tropics with global public health ramifications. It is estimated that nearly half of the worlds’ population is at risk of malaria and with estimated 450,000 deaths caused by malaria in 2019 [1]. Majority of malaria transmission and infections occur in sub-Saharan African. Non-endemic regions such as Europe, Canada and the USA are thought to acquire malaria from endemic areas mainly during a travel visit for pleasure or business [2]. Many of these imported malaria infections are caused by *Plasmodium falciparum* and originate from Africa. Infections with other Plasmodium species such as *P. malariae, P. vivax* and *P. ovale* are also known to occur [3]. It is important that these infections are promptly detected and treated as most returning travelers are immunologically naïve with little or no immunity, thus early treatment is critical to protect them from severe malaria and death.

In most countries, the diagnosis of malaria cases is performed using microscopy or the country’s approved malaria rapid diagnostic test (RDT). The accuracy of malaria microscopy in non-endemic countries is often compromised by the inexperience of many of the technicians given that many do not get to see parasites often, this is especially true for non-falciparum infections [3]. A common alternative to microscopy is the use of *P. falciparum*-specific histidine-rich protein (HRP)2-based RDTs combined with a pan *Plasmodium* antigen (typically lactate dehydrogenase or aldolase) for diagnosis. However, while these RDTs can accurately detect *P. falciparum*, they are not capable of distinguishing between the non-*falciparum* species. Therefore, alternative diagnostic tests such as nucleic-acid tests (molecular tests) may be more appropriate to diagnose imported malaria cases due to their high sensitivity and ability to distinguish different malaria species.

Recently, the *illumigene* (now Athelia)-malaria loop mediated isothermal amplification (LAMP) tests have been utilized in Canada and reported to have high sensitivities and specificities [4]. The *illumigene* malaria LAMP assay has also been utilized to diagnose suspected imported malaria cases in several European laboratories, with great success [3–6]. The malaria LAMP test was shown to have similar sensitivities and specificities to the traditional polymerase-chain reaction (PCR)-based assay but was both easier to perform and cost effective, making the LAMP platform a viable option for use in the diagnosis of imported malaria cases.

Returning travelers and visitors from malaria endemic countries contribute to an average of 1,500 malaria confirmed cases annually in the USA [7]. Malaria diagnosis in the USA is conducted at the local health care facilities or with the support of local and state health departments, often using microscopy and/or RDTs. Suspected malaria cases are determined by a positive RDT, which then require a confirmation by microscopy or PCR to be classified as a confirmed case [8]. Most primary health facilities do not have the ability to do microscopy and in this cases, RDTs are frequently used. The only RDT approved for clinical use in the USA is the BinaxNOW RDT, which has been reported to have low performance compared to other RDTs [9]. Therefore, a sensitive tool such as LAMP has potential use for rapid detection of malaria parasites in health care settings. In this context, no studies to date have been undertaken to evaluate the use of the Alethia® Malaria Plus LAMP for the diagnosis of imported malaria in the USA. We evaluated the test accuracy of the Alethia® Malaria Plus LAMP assay using a real-time PCR assay as a reference test.

## Main text

### Methods

#### Clinical samples

A total of 95 retrospective clinical samples were available for use in this study. These were pre-treatment blood samples from patients presenting with symptoms suggestive of malaria after returning to the USA from visits to malaria endemic countries and subsequently submitted to the CDC by clinicians and/or state public health departments for confirmatory malaria diagnosis. Whole blood samples arrived at ambient temperature in EDTA tubes. DNA was extracted from all samples using the QIAamp DNA Mini Kit (Valencia, CA) following the manufacturer's recommendations. All samples were first tested with a previously described real-time photo-induced electron transfer PCR (PET-PCR) [10] assay for *Plasmodium* and the four human infecting malaria species: *P. falciparum, P. vivax, P. ovale,* and *P. malariae*.

#### PET-PCR assay

All PET-PCR assays were performed in a 20 µl reaction mix containing 2 × TaqMan Environmental Master Mix 2.0 (Applied BioSystems, Forest City, CA, USA) and 5 µl of DNA template, as previously described [10]. The following cycling parameters were used: initial hot-start at 95 °C for 15 min, followed by 45 cycles of denaturation at 95 °C for 20 s, annealing at 63 °C for 40 s and an extension at 72 °C for 10 s. A cycle threshold (Ct) cut off was set at 40: Ct values above 40 were considered negative, while values below 40 were considered positive.

#### Alethia® Malaria Plus LAMP assay

The Alethia® Malaria Plus LAMP assay is a *Plasmodium* genus-specific assay which utilizes a centrifuge-free gravity-driven gel filtration column for DNA extraction and ambient-stable amplification reagents (DNA polymerase, deoxynucleotide triphosphates, primers) and supplies. The test device consists of two-chambers/tubes containing the lyophilized amplification reagents of *Plasmodium* sp. primers (TEST Chamber) or human mitochondrial DNA-specific primers (CONTROL Chamber). The amplification is performed using the Alethia® Malaria Plus LAMP incubator and reader according to manufacturer’s instructions. The results are displayed on a LCD screen as “positive” or “negative”.

A total volume of 50 µl of previously frozen peripheral blood was mixed with 320 µl assay lysis buffer and the lysate was transferred to the gel-filtration column according to manufacturer’s instructions. Sample elutions, containing the DNA, were collected and 50 µl directly added to the TEST and CONTROL chambers/tubes of the Alethia® Malaria Plus LAMP test device.

#### Data analysis

Results (Ct values for PET-PCR and positive/negative for LAMP) were entered into Microsoft Excel (Microsoft Office 365 ProPlus) and LAMP test performances (sensitivity) and Cohen Kappa test for agreement between the LAMP and PET-PCR were determined using the free version of MedCalc statistical software.

### Results

From the 95 samples processed, the PET-PCR assay identified 90 malaria positive and 5 negative samples. The Alethia® Malaria Plus LAMP also identified the same 90 positive samples and 5 negative samples giving a kappa score of 1 (Cohen’s kappa test), indicating a perfect agreement. The sensitivity of the Alethia® Malaria Plus assay was 100% (95% confidence interval: 96 to 100%), Table 1. The *Plasmodium* species were determined using the PET-PCR as shown in Table 1. The species could not be determined for 2 of the 90 *Plasmodium* positive samples and were reported as *Plasmodium*, Table 1.

**Table 1:** Comparative results between PET-PCR and Alethia® Malaria Plus LAMP

Molecular Platforms	Species identified									
	*P. falciparum (Pf)*	*P. vivax (Pv)*	*P. ovale (Po)*	*P. malariae (Pm)*	*Pf/Pm*	*Pf/Po*	*Pf/Pv*	*Negative*	*Plasmodium*	*Total*
Reference PET-PCR	66	5	8	6	1	1	1	5	2	95
Alethia® Malaria Plus LAMP	*	*	*	*	*	*	*	5	90	95

^*^ Alethia® Malaria Plus is a Plasmodium genus-specific assay

Initially, 4 of the 95 samples tested (4.2%) with the Alethia® Malaria Plus LAMP assay gave irregular results: “Invalid” (2 samples) and “Empty” (2 samples). As per the manufacturer’s instructions, these results are not reportable results and are likely due to inhibitory specimen, improper sample preparation, reagent failure, instrument failure or internal control failure, dirty device or improperly seated device. The manufacturer recommend that the tests are repeated using the original sample. Therefore, these samples were retested in a similar fashion as the initial test, as per the manufacturer’s recommendations. The four samples gave valid results upon repeating the test one time.

The maximum time to results, from sample preparation (DNA extraction) to obtaining results, when using the Alethia® Malaria Plus LAMP test was 1 h in contrast to more than 2 h required to complete the PET-PCR assay, Table 2.

**Table 2:** Time required to obtain results

Technique	Sample preparation (minutes)	Assay running (minutes)
Alethia® Malaria Plus LAMP	15–20	40
PET-PCR	40–60	95

### Discussion

In this study, we demonstrate the utility of the Alethia® Malaria Plus LAMP assay for the accurate and rapid detection of malaria parasites in specimens from returning travelers in the USA. The Alethia® Malaria Plus LAMP is the second of the LAMP based assays that is commercially available for the detection of malaria [11–14]. Our evaluation of the Alethia® Malaria Plus LAMP showed a 100% sensitivity when compared to our in-house PET-PCR reference test. Specificity could not be accurately determined due to the low number of negative samples but, Alethia® Malaria Plus identified all five negative samples correctly. This is in agreement with several studies that have demonstrated high specificities and sensitivities for this assay in detecting imported malaria cases [3, 4, 6, 15]. Studies conducted in Canada [15], Belgium [16] and Germany [5, 6] all demonstrated high sensitivity (range 97.3–100%) and specificity (range 91.5–100%) for this LAMP test. In addition, a study conducted in Senegal, a malaria endemic country, found the assay to have 97% sensitivity and 88% specificity [17].

Although the Alethia® Malaria Plus LAMP assay performed well, with exceptional sensitivity, faster turnaround time and ease of use compared to conventional and real-time PCR, it is not without drawbacks. The occasional irregular results which, as per the manufacturer’s instructions, are likely due to a wide range of factors including inhibitory specimen, improper sample preparation, reagent failure, instrument failure or internal control failure, dirty device and improperly seated device appeared to be a random occurrence. Because this requires that the testing is repeated with a fresh sample, it adds to the time required to get a result, leading to delays in obtaining the results or no testing if additional sample is unavailable. This was not unique to the testing in this study, as previous investigators made similar observations [4, 15]. If unresolved, these irregular results would necessitate additional testing with other malaria molecular diagnostic tests leading to delays in making a diagnosis. Another drawback, as highlighted and discussed by previous investigators, is the fact that this assay is a genus-specific assay and therefore requires that further testing is undertaken in order to determine the infecting *Plasmodium* species. While this does not matter if the treatment regime is the same irrespective of the infecting species, it matters in countries where treatment is different depending on the species and where radical cure of hypnozoites that cause relapse malaria, associated with *P. vivax* and *P. ovale,* is recommended. In the USA, radical cure for *P. vivax* and *P. ovale* infections is recommended with either primaquine phosphate or tafenoquine (Krintafel™) [18].

Despite the limitations, a major benefit of this assay is the fact that it offers a much needed malaria screening test for use in non-endemic countries including the USA. This genus-specific assay can be utilized as an initial screening test for all cases suspected as malaria after which patients can be correctly triaged depending on the LAMP results. Indeed, Rypien et al. [4] proposed a screening scheme in which the Alethia® Malaria Plus LAMP assay is utilized to screen for *Plasmodium* positive specimen. No further malaria testing would be required for the LAMP negative specimens, eliminating a large number of negative samples that would otherwise be tested by microscopy, RDTs or other molecular tests. Follow up testing would therefore be limited to the LAMP positive specimens only which are likely to be few in non-endemic countries. Such a testing scheme was reported to have a cost–benefit of reagents and labor leading to savings of up to USD 13 per tests [15].

The Alethia® Malaria Plus LAMP assay is sensitive and easy to perform and can provide an alternative test of choice for the accurate screening of malaria parasites in non-endemic countries such as the USA.

## Limitations

Our study was limited in that we used available retrospective specimens which were limited in number and did not give us the opportunity to test the accuracy of this test in detecting low-parasite density infections and we could not accurately access the specificity of the assay given the limited number of negative samples In addition, we did not undertake reproducibility analysis for this assay.

## Data Availability

The datasets used and/or analyzed during the current study are available from the corresponding author on reasonable request.

## Abbreviations

Ct: Cycle threshold
HRP-2: Histidine-rich protein-2
LAMP: Loop mediated isothermal amplification
EDTA: Ethylenediaminetetraacetic acid
PCR: Polymerase chain reaction
PET: Photo-induced electron transfer
RDT: Rapid diagnostic test
USA: United States of America
USD: USA Dollars
